# Management of Large Splenic Artery Pseudoaneurysm Presenting with Gastrointestinal Bleeding

**DOI:** 10.7759/cureus.100299

**Published:** 2025-12-28

**Authors:** Dionysios Prevezanos, Dimitrios Vlachos, Michail Konstantinidis, Konstantinos Kossenas, Nikolaos Machairas

**Affiliations:** 1 2nd Department of Propaedeutic Surgery, Laiko General Hospital of Athens, Athens, GRC; 2 Renal Transplantation Unit, Laiko General Hopsital of Athens, Athens, GRC; 3 Medicine, University of Nicosia, Nicosia, CYP; 4 Hepato-Pancreatico-Biliary Surgery, Royal Free Hospital, London, GBR

**Keywords:** abdominal vascular complications, chronic pancreatitis, endovascular embolization, gastrointestinal bleeding, splenic artery pseudoaneurysm

## Abstract

Splenic artery pseudoaneurysms are rare but life-threatening complications of chronic pancreatitis (CP). Their management often requires a tailored approach to address the vascular abnormality while minimizing patient morbidity.

We report the case of a 59-year-old Caucasian female patient with a history of chronic alcohol consumption who presented to the Emergency Department at Laiko General Hospital of Athens with complaints of melena and diffuse abdominal pain. An endoscopy revealed a Forrest 2b lesion and a pulsatile bulge at the posterior stomach wall. Due to the pulsatile bulge, the lesion was not intervened upon during endoscopy, as it was suspected to represent an underlying vascular abnormality. Initially presumed to be an ulcer secondary to chronic alcohol use, the lesion prompted further evaluation with a contrast-enhanced CT scan. Imaging confirmed a splenic artery pseudoaneurysm measuring 4.09 x 4.58 cm and revealed evidence of CP. Endovascular embolization of the pseudoaneurysm was performed, achieving complete occlusion and leading to a favorable outcome. The patient’s postoperative course was uneventful, with stable laboratory findings and functional splenic tissue on follow-up imaging. Over a six-month follow-up, serial imaging confirmed the absence of relapse or complications.This case underscores the importance of individualized care and highlights the role of interventional radiology as a primary modality for managing pseudoaneurysms without hemorrhage. A minimally invasive approach can achieve effective resolution while preserving splenic function.

## Introduction

Gastrointestinal (GI) bleeding is a common and potentially life-threatening clinical presentation, accounting for a significant proportion of emergency department admissions worldwide [1]. While the majority of cases are attributed to peptic ulcer disease, less frequent vascular etiologies may pose substantial diagnostic and therapeutic challenges. Failure to promptly recognize these uncommon causes can result in delayed treatment and increased morbidity and mortality [2]. 

Chronic pancreatitis (CP) is a progressive inflammatory disease of the pancreas, characterized by recurrent episodes of inflammation that lead to irreversible replacement of pancreatic parenchyma with fibrous connective tissue. While the most common etiological factors include excessive alcohol consumption and smoking, a significant proportion of cases remain idiopathic [3]. As a progressive disease, CP often requires imaging for diagnosis and monitoring, with CT being regarded as the gold-standard modality [4]. CP is associated with a marked reduction in quality of life and an increased mortality rate, estimated to be 3.6 to 4 times higher compared to the general population [5,6].

The clinical manifestations of CP vary, with abdominal pain being the most common symptom. Less frequent symptoms include fever, early satiety, and weight loss [7]. Complications of CP can involve both local and systemic effects, including pancreatic infection, pseudocyst formation, benign biliary strictures, pancreatic fistulas, gastric outlet obstruction, as well as vascular complications such as venous thrombosis and visceral artery pseudoaneurysms [8-11].

Among the vascular complications, visceral artery pseudoaneurysms represent a rare but potentially life-threatening condition. These pseudoaneurysms occur as a result of chronic inflammation, fibrosis, and enzymatic digestion of the arterial walls. Splenic artery pseudoaneurysms are the most common type of visceral artery pseudoaneurysms, accounting for 60-70% of cases. In patients with CP, the prevalence of splenic artery pseudoaneurysms is reported to be as high as 21% [12]. These lesions may present with GI bleeding and mimic more common causes, potentially leading to diagnostic delay or inappropriate endoscopic intervention. Awareness of this presentation is crucial, as early cross-sectional imaging or angiographic evaluation enables timely diagnosis and facilitates definitive minimally invasive management.

Although splenic artery pseudoaneurysms associated with CP have been described in case reports and small case series, their presentation as upper GI bleeding remains uncommon and diagnostically challenging. Reports highlight variable clinical manifestations and emphasize the importance of maintaining a high index of suspicion, particularly when endoscopic findings are atypical or inconclusive [12].

In this report, we describe a rare and critical case of a patient with CP complicated by a splenic artery pseudoaneurysm identified with significant GI bleeding. We aim to provide a detailed account of the clinical presentation, diagnostic process, and therapeutic strategy employed in the management of this condition.

## Case presentation

A 59-year-old Caucasian female patient presented to the Emergency Department at Laiko General Hospital of Athens with complaints of black, tarry stools and diffuse abdominal pain, which were clinically assessed as melena. Her medical history was significant for chronic alcohol consumption for the past 20 years, estimated at approximately one bottle of wine per day. There was no prior history of GI bleeding, previously diagnosed CP, anticoagulant or antiplatelet therapy, or regular nonsteroidal anti-inflammatory drug use. At presentation, her hemoglobin level was 6.8 g/dL. Despite severe anemia, the patient remained hemodynamically stable, and blood transfusion was deferred following initial stabilization, close monitoring, and absence of ongoing active bleeding.

Initial emergency upper GI endoscopy revealed a gastric ulcer with an adherent clot and no signs of active bleeding, as well as a pulsatile bulge on the posterior gastric wall in close proximity to the ulcerated area, raising concern for an underlying vascular lesion. Owing to the pulsatile nature of the lesion and concern for an underlying vascular abnormality, no endoscopic intervention was attempted to avoid the risk of catastrophic hemorrhage. Initially, the lesion was presumed to represent a gastric ulcer related to chronic alcohol use.

**Figure 1:**
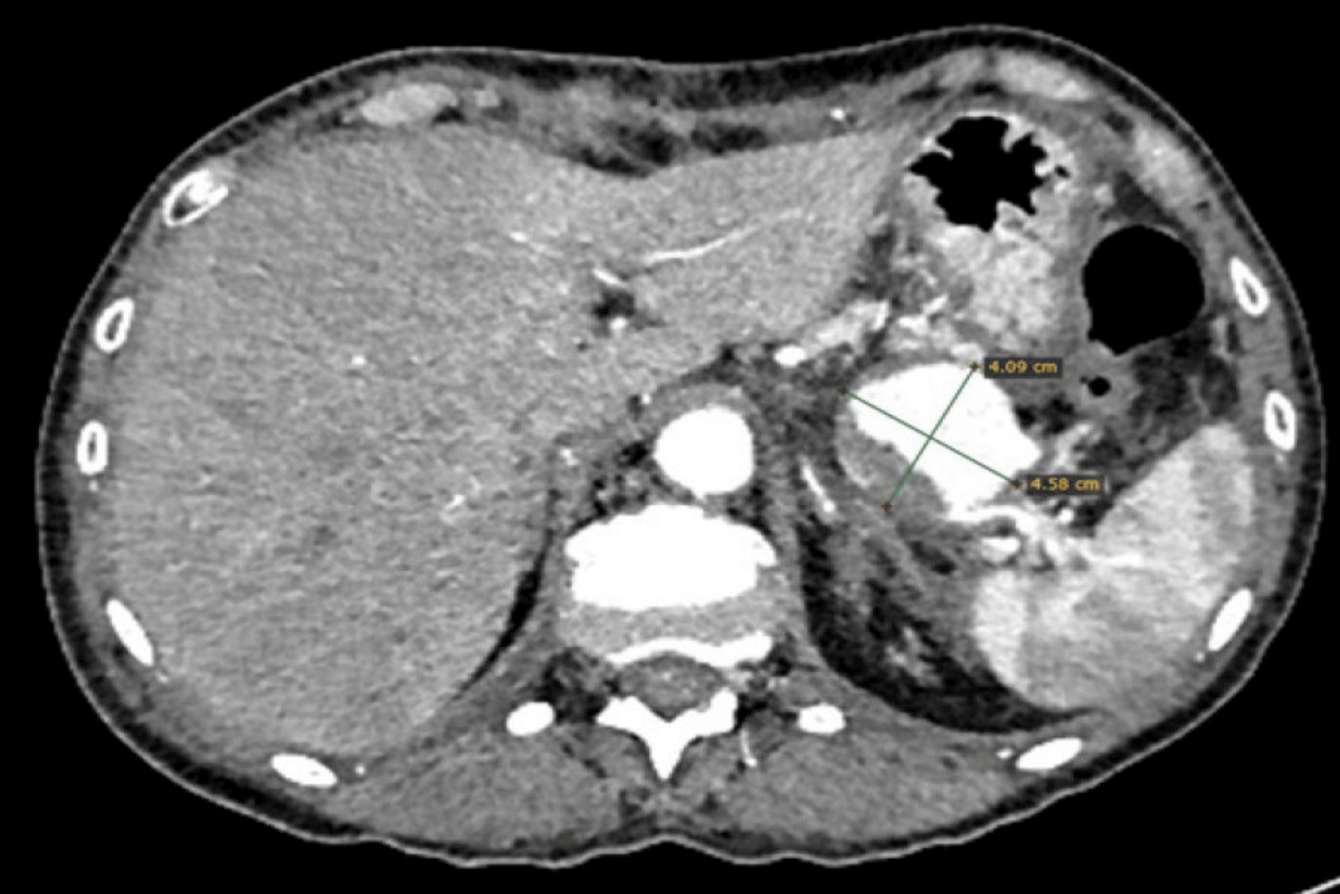
CTA demonstrating a pseudoaneurysm of the distal splenic artery. Multiple peripheral splenic infarcts are evident. CTA: CT angiography

**Figure 2:**
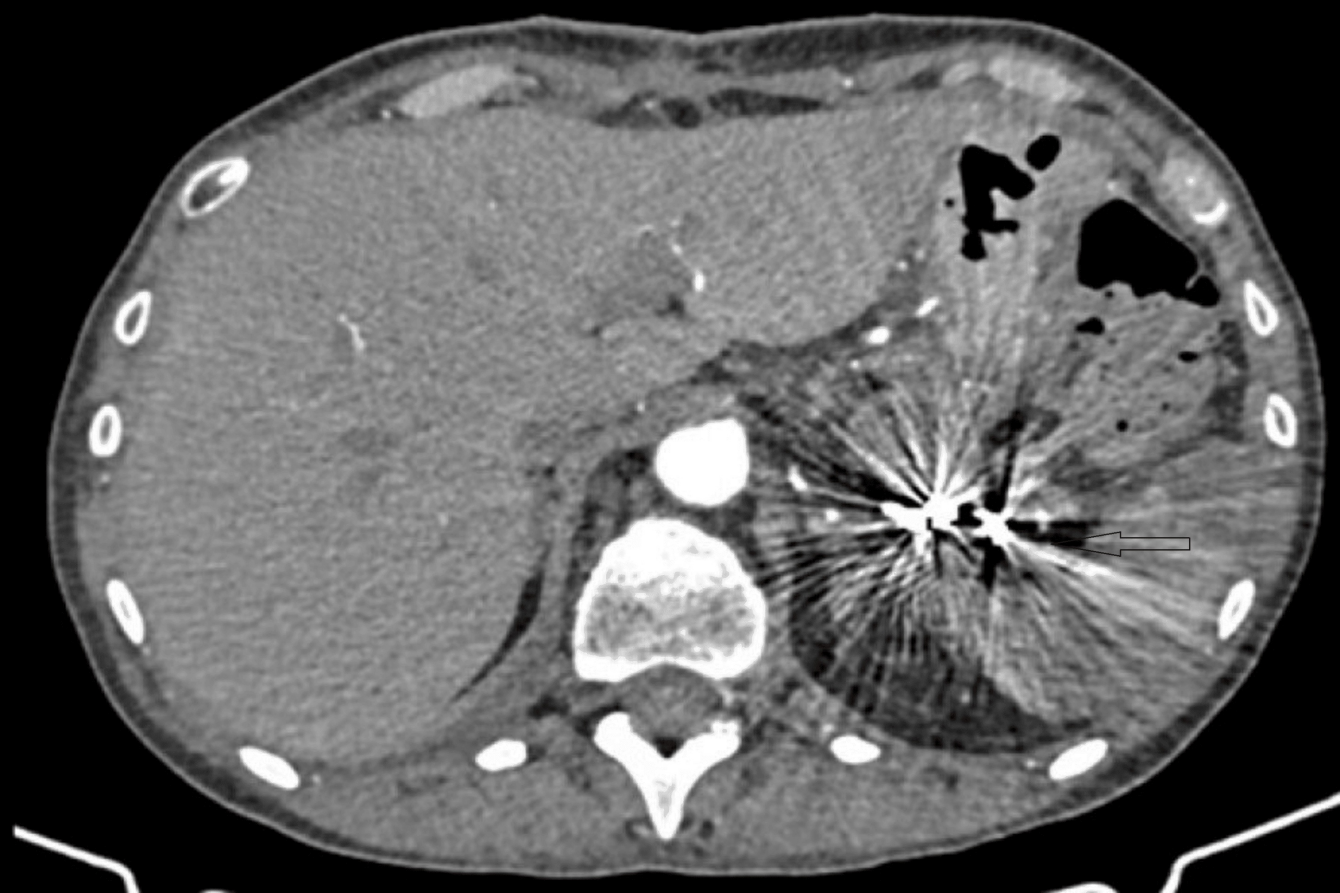
CTA performed after the embolization of the splenic artery, demonstrating total occlusion of the pseudoaneurysm CTA: CT angiography

To further evaluate the pulsatile lesion, a contrast-enhanced CT scan (CT angiography (CTA)) was performed. Imaging confirmed the presence of a splenic artery pseudoaneurysm measuring 4.09 × 4.58 cm and revealed radiologic features consistent with CP (Figures 1, 2).

Following a multidisciplinary team discussion, a decision was made to manage the pseudoaneurysm with endovascular embolization. The patient underwent the procedure on the same day. After selective catheterization of the splenic artery, a sandwich coil embolization technique was employed, with multiple coils placed both proximally and distally to the pseudoaneurysm. Completion angiography confirmed complete exclusion of the pseudoaneurysm from the systemic circulation.

**Figure 3:**
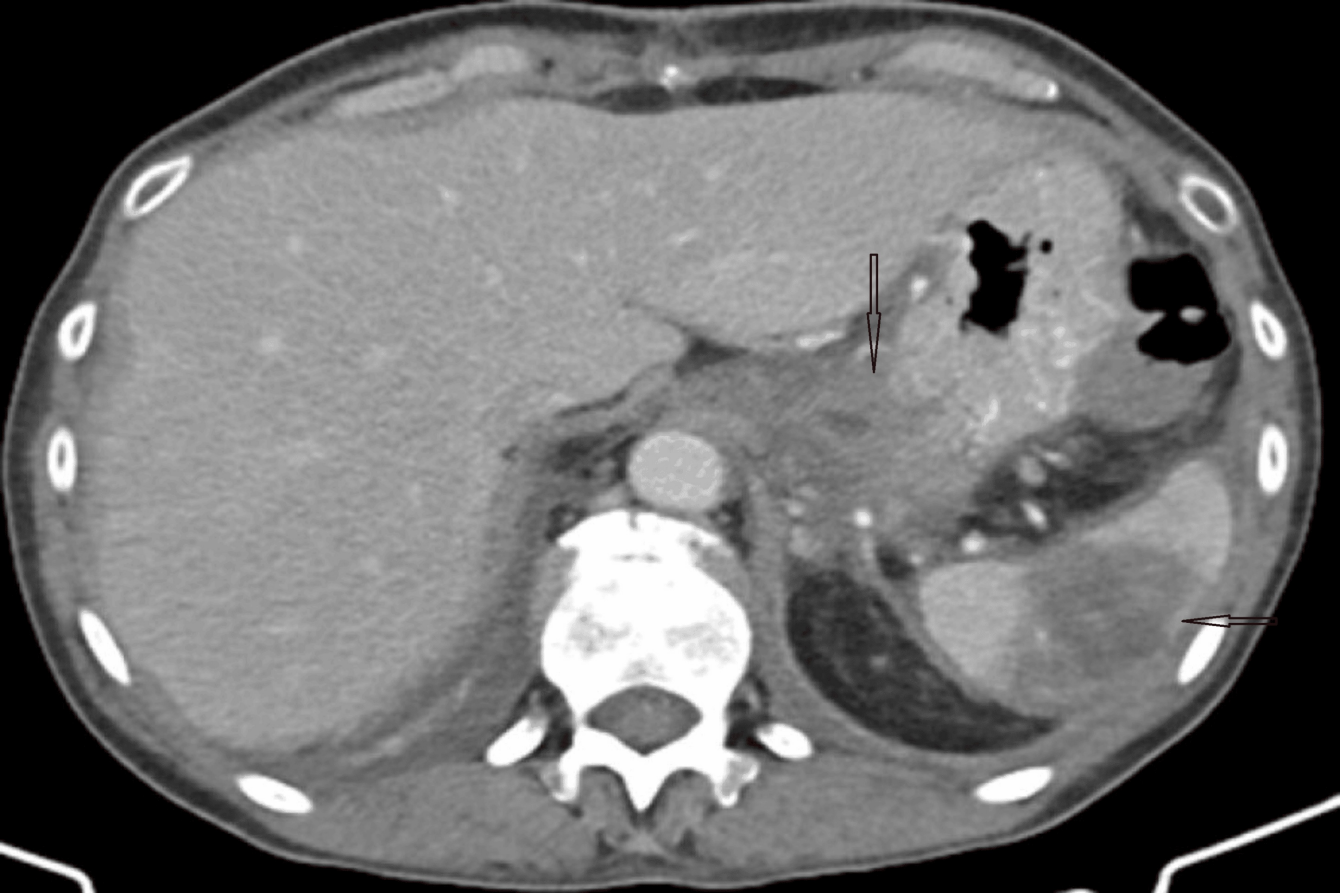
Central splenic infarct with viable peripheral splenic tissue post embolization

Postprocedurally, the patient had an uneventful recovery. She remained afebrile and hemodynamically stable, with gradual improvement in her abdominal symptoms. Follow-up imaging performed five days later demonstrated successful exclusion of the pseudoaneurysm, evidenced by the absence of blood flow within it. Partial splenic infarction was noted without clinical sequelae, with preserved overall splenic function; this finding is illustrated in Figure 3.

The patient was discharged in stable condition seven days after embolization and was closely monitored over a six-month follow-up period. Serial imaging and laboratory tests revealed no evidence of relapse or new complications. Follow-up imaging demonstrated a gradual reduction in the size of the pseudoaneurysm. The patient experienced no recurrent symptoms, and splenic function remained preserved (Figure 3).

## Discussion

Splenic artery aneurysms (SAAs) and pseudoaneurysms are rare but significant vascular complications often associated with CP. The spread of proteolytic fluids from inflamed pancreatic tissue weakens arterial walls, leading to the formation of pseudoaneurysms [13]. Most patients with splenic artery pseudoaneurysms are asymptomatic, but approximately 20% may experience symptoms such as abdominal pain, just like in our case. These vascular abnormalities are often detected incidentally during imaging [13]. Pseudoaneurysms most commonly involve the splenic artery, followed by gastroduodenal, pancreaticoduodenal, gastric, and hepatic arteries [14]. GI bleeding caused by splenic artery pseudoaneurysms is rare, but several cases have been reported [15].

Imaging modalities play a crucial role in the diagnosis, characterization, and management planning of SAAs. Multidetector CTA (MDCTA) has emerged as the gold standard due to its high spatial and temporal resolution, providing detailed visualization of the aneurysm’s size, shape, location, and relationship with surrounding structures. Postprocessing techniques, such as volume rendering (VR), maximum intensity projection (MIP), and curved planar reconstructions (CPR), enhance the diagnostic accuracy and aid in preoperative planning [16].

While ultrasound is a widely accessible initial modality, it is limited by operator dependency, bowel gas interference, and lower sensitivity for small aneurysms [17]. Magnetic resonance angiography (MRA) is a viable alternative in cases where iodinated contrast for MDCTA is contraindicated, although it is less practical for emergencies due to longer acquisition times and higher costs [18]. Historically, digital subtraction angiography (DSA) was considered the gold standard for vascular imaging but is now primarily reserved for interventional procedures due to its invasive nature. These advanced imaging technologies not only improve diagnostic confidence but also allow for tailored interventions, such as endovascular treatments or surgical approaches, making imaging an indispensable component of SAA management.

No universally accepted guidelines are available for the management of SAA. Endovascular interventions have emerged as the first-line treatment for pseudoaneurysms due to their minimally invasive nature, high success rates, and low morbidity [19]. Techniques such as coil embolization, glue embolization, and thrombin injection directly into the pseudoaneurysm are commonly used [20]. However, complications including coil migration, splenic infarction, or aneurysm recanalization may necessitate additional interventions. Partial splenic infarction is a known and expected finding following splenic artery embolization and was observed in our patient without clinical sequelae. The patient was closely monitored for signs of clinically significant splenic ischemia or rebleeding, with preservation of peripheral splenic perfusion on follow-up imaging.

Surgical intervention remains essential in cases where endovascular approaches fail or when pseudoaneurysms involve significant adjacent structures. Procedures like aneurysmectomy with splenectomy or distal pancreatectomy are often employed, particularly in cases involving fistulas. For asymptomatic pseudoaneurysms under 2 cm, conservative management with close radiological monitoring may be sufficient [20].

## Conclusions

This case highlights that GI bleeding may not always be attributable to the most apparent or common etiology. What initially appeared endoscopically as a gastric ulcer was ultimately caused by a splenic artery pseudoaneurysm, underscoring the importance of maintaining diagnostic vigilance and avoiding premature diagnostic closure. Endoscopic vascular warning signs, such as a pulsatile submucosal bulge, should raise suspicion for an underlying vascular lesion and prompt early cross-sectional imaging. Beyond definitive treatment, timely recognition, a high index of suspicion, and multidisciplinary coordination are critical to prevent catastrophic hemorrhage and to enable safe, minimally invasive management while preserving splenic function.
